# Evaluation of Phage Display Discovered Peptides as Ligands for Prostate-Specific Membrane Antigen (PSMA)

**DOI:** 10.1371/journal.pone.0068339

**Published:** 2013-07-25

**Authors:** Duanwen Shen, Fei Xie, W. Barry Edwards

**Affiliations:** 1 Radiology, Washington University, St. Louis, Missouri, United States of America; 2 Radiology, University of Pittsburgh, Pittsburgh, Pennsylvania, United States of America; Florida International University, United States of America

## Abstract

The aim of this study was to identify potential ligands of PSMA suitable for further development as novel PSMA-targeted peptides using phage display technology. The human PSMA protein was immobilized as a target followed by incubation with a 15-mer phage display random peptide library. After one round of prescreening and two rounds of screening, high-stringency screening at the third round of panning was performed to identify the highest affinity binders. Phages which had a specific binding activity to PSMA in human prostate cancer cells were isolated and the DNA corresponding to the 15-mers were sequenced to provide three consensus sequences: GDHSPFT, SHFSVGS and EVPRLSLLAVFL as well as other sequences that did not display consensus. Two of the peptide sequences deduced from DNA sequencing of binding phages, SHSFSVGSGDHSPFT and GRFLTGGTGRLLRIS were labeled with 5-carboxyfluorescein and shown to bind and co-internalize with PSMA on human prostate cancer cells by fluorescence microscopy. The high stringency requirements yielded peptides with affinities K_D_∼1 µM or greater which are suitable starting points for affinity maturation. While these values were less than anticipated, the high stringency did yield peptide sequences that apparently bound to different surfaces on PSMA. These peptide sequences could be the basis for further development of peptides for prostate cancer tumor imaging and therapy.

## Introduction

Prostate cancer (PCa) is the second leading cause of death in men in the United States and is the most diagnosed cancer in men. If diagnosed early, the five year survival rate is very good. The implementation of prostate specific antigen (PSA) screening, which facilitates earlier detection of prostate cancer, is partly responsible for the decrease in mortality [1], [2]. However, PSA levels are not an accurate predictor of the aggressiveness of prostate cancer, with the PSA rate of change (PSA velocity) not able to discriminate between men with cancer and those with negative biopsies [3], [4], [5]. On the contrary, there are high frequencies of prostate-specific membrane antigen (PSMA) overexpression in all stages and grades of PCa patients. The overexpression of PSMA was significantly associated with tumor stage, where the higher Gleason graded tumors corresponded with higher expression levels of PSMA [6], [7]. Therefore, PSMA is an alternative to PSA as a diagnostic biomarker for detection of PCa [6].

PSMA is a 100-kDa transmembrane glycoprotein with folate hydrolase activity (hydrolysis of the terminal glutamates from γ-linked polyglutamates) as well as NAALADase activity (hydrolysis of the terminal glutamate from the neuropeptide NAAG), which was found not only in normal prostate epithelium but also in the central nervous system and the proximal gastrointestinal tract [8]. PSMA is also overexpressed in the neovascular endothelium of most solid tumors, including lung, colon, breast, renal, transitional cell, and pancreas cancers [9], [10]. However, it is not expressed in normal vasculature [10], [11]. Interestingly, the average serum PSMA value for prostate cancer was significantly higher (*P*<0.001) than that for benign prostate hyperplasia and the normal groups [7]. Recently, a radiolabeled monoclonal antibody (MAb) to PSMA named J591, was used for non-invasive imaging and radiotherapy for prostate cancer in human subjects with positive results [12], [13], [14]. PSMA on the cell surface is internalized by J591 [15] and endocytosed through clathrin coated pits, though the natural ligand of PSMA is unknown. Therefore PSMA targeting can play an important role in directing the treatment and therapeutic options for patients with prostate cancer.

Monoclonal antibodies may appear to be ideal imaging agents because of their high specificity and affinity for the target epitopes, but their most serious shortcoming is their very long plasma half-lives which ultimately set optimal signal to noise at days post administration. For example, the optimal imaging time of ^111^In-Mab 7E11 is 96 h post-administration [16]. For this reason, low molecular weight ligands are sought for their more favorable pharmacokinetics. Small hydrophilic inhibitors of the enzymatic domain of PSMA have been either labeled with ^18^F and ^68^Ga for molecular imaging of PSMA by positron emission tomography or with ^99m^Tc single photon emission computed tomography [17], [18], [19], [20]. Additionally, these potent inhibitors have been labeled with near infrared dyes for optical imaging [21], [22]. All of the analogs have exhibited PSMA-mediated tumor uptake in animal models with low non-target tissue uptake except for kidney.

As of yet, however, there have been no reported peptides of high affinity that are suitable for PSMA imaging. Peptides have displayed pharmacokinetics suitable for imaging patients on the same day as administration with good target tissue uptake and low non-target tissue uptake. (reviewed in [23]) Unlike small molecule inhibitors, peptides are biopolymers that are easily accessible by solid-phase synthesis from readily available starting materials. Furthermore, they are generally structurally more robust than antibodies and are suitable surrogates with affinity sufficient for molecular imaging. Consequently the discovery of peptides with high affinity for PSMA would be of benefit for anti-prostate cancer research.

One way of finding peptides for a ligand is through panning of a phage-displayed peptide library for the ligand of interest. Phage display is a technique where random peptides are fused to the coat protein of a bacteriophage in a manner that makes them accessible to target ligands. The DNA encoding the peptide sequence is protected within the virion [24]. Panning is a technique of successive binding, rinsing, and elution of specifically bound phage to target receptors. This will result in enrichment of peptide-ligands with stronger affinity for the target receptor.

The goal of this research was to find lead peptide sequences that bind PSMA for further development as imaging agents for targeted therapy by screening a library of phage displayed peptides against PSMA. A 15-mer library was screened against the soluble ectodomain of PSMA. In the initial step, the library was pre-cleared by intravenous administration in a mouse to remove phages that bound to vascular proteins thus clearing the phage pool of non-specific binders. The resulting library was then screened against the soluble ectodomain of PSMA under low stringency conditions for two rounds of affinity based selection followed by one round of high stringency selection. The purpose of the high stringency selection was to direct selection toward high-affinity binders. Nine unique sequences were determined from twelve isolated phage clones. These sequences were tested for specificity toward PSMA expressed on human prostate cancer cells and the binding affinity was quantified by AlphaScreen and PSMA enzyme assays.

## Materials and Methods

### Materials

F3-15mer random bacteriophage library and K91BluKan (B91BK) *E.Coli* expression system was depleted of phage that bind proteins of mouse vasculature as previously described [25]. His-tagged PSMA (PSMA-His_6_) was purchased from R&D Systems. Rabbit monoclonal anti-PSMA antibody was purchased from Epitomics (Burlingame, CA). Biotinylated rabbit polyclonal anti-Fd bacteriophage antibody was purchased from Sigma-Aldrich (St. Louis, MO). Alexa 488 conjugated streptavidin was purchased from Invitrogen (Carlsbad, CA). TRITC labeled donkey anti-Rabbit IgG was purchased from Jackson ImmunoResearch Laboratories, INC. (West Grove, PA). TBS is 50 mM Tris, 150 mM NaCl, pH 7.4. Fmoc-L-Amino acids, HCTU (2-(6-Chloro-1H-benzotriazole-1-yl)-1,1,3,3-tetramethylaminium hexafluorophosphate), and Cl-HOBt (6-Chloro-1-hydroxybenzotriazole) were purchased from AAPPTec (Louisville, KY). The Rink amide AM resin LL (100–200 mesh) and the biotin-PEG NovaTag resin were purchased from EMD Millipore (Billerica, MA). The Fmoc_4_-Lys_2_-Lys-β-Ala Wang resin and the N-Fmoc-Amido-dPEG_6_ acid were purchased from Peptide International (Louisville, KY). The 3-maleimido-propionic acid was purchased from Chem-Impex International (Wood Dale, IL). The 5-FAM (5-carboxyfluorescein) was purchased from AnaSpec (Fremont, CA). The AlphaScreen Histidine detection kit which contains the streptavidin donor beads and the nickel chelate acceptor beads was purchased from PerkinElmer (Waltham, MA). All other reagents were purchased from Sigma Aldrich (St. Louis, MO).

### General methods

All peptides were synthesized on the CEM Liberty1 microwave peptide synthesizer (Matthews, NC) using the Rink amide resin, except for the biotinylated peptides which used the biotin-PEG NovaTag resin. 5-FAM peptides were synthesized by coupling 5-FAM to the N-terminus of the peptide on resin. All peptides were purified by HPLC and the molecular weights were confirmed by either ESI MS (Waters LCT Premier XE) or MALDI-TOF MS (Applied Biosystems Voyager DE-STR).

### Tetrameric peptide synthesis

The tetrameric peptide was synthesized as previously described [26]. Briefly, the thiol containing reactive peptide monomer was synthesized by introducing a GGGS-(PEG)_6_-C linker to the C-terminus of peptide 562 (Table 1). The maleimide-activated core was synthesized by coupling the 3-maleimido-propionic acid on the Fmoc_4_-Lys_2_-Lys-β-Ala Wang resin. The purified reactive peptide monomer and the maleimide-activated core were conjugated in PBS with the final tetrameric peptide purified through HPLC.

**Table 1:** Sequences of phage clones isolated after 3-round of panning and IC_50_ values of the peptides analyzed.

Clone ID	Sequence	#	AlphaScreen	Enzyme inibition
B	LPIFKVDF*GDHSPFT*	nd	nd	nd
C	ARMFLLFLMRCIGCY	565*	>4.83 µM	a
D, H	***SHSFSVGSG*** *DHSPFT*	562	nd	661 µM (76 µM)†
	***SHSFSVGSG*** *DHSPFT*	(562)_4_	nd	311 µM
E, F	***SHSFSVGSG***SGDHSP	nd	nd	nd
G	LSFFSCWLRRSFSLT	564*	1.72 µM	b
I	***EVPRLSLLAVFL***VVM	nd	nd	nd
J	***EVPRLSLLAVFL***CNG	nd	nd	nd
K, L	***EVPRLSLLAVFL***VAN	nd	nd	nd
M	GRFLTGGTGRLLRIS	563	708 nM	c

* Cys(Acm) in peptides 564 and 565.

† Ki value from the Cheng-Prussof equation.

a , no inhibition observed at 14.5 µM.

b , no inhibition observed at 55.5 µM.

c , no inhibition observed at 1 mM.

nd, not determined.

### Screening the phage library for PSMA-targeted peptides

Standard preparations and procedures methods were described from George P. Smith on the website http://www.biosci.missouri.edu/SmithGP/PhageDisplayWebsite/PhageDisplayWebsiteIndex.html) and as previously described [27]. To deplete the library of background binding phage, the plate was coated with monoclonal anti-polyhistidine antibody (mouse), then 5% bovine serum albumin (BSA) without PSMA-His_6_. Then, 1.9×10^13^ colony-forming units (cfu) were added and allowed to bind (ambient temperature, 1 h); the non-binding phage were collected, amplified and purified for the first round of screening.

All screening began with 1.9×10^13^ cfu and was carried out in one well of a 6-well plate (Corning, binding capacity 2.5 µg IgG/well) in a volume of 1.5 mL. The well was coated with mouse monoclonal anti-polyhistidine antibody (1.5 mL, 5 µg/mL) followed by blocking (5% BSA, TBS, 3 h). PSMA-His_6_ was then captured by the immobilized antibody (3 h, ambient temperature). For rounds one and two of panning (18 h, 4**°**C), low stringency was used (42 nM PSMA-His_6_). In the third round of screening, the stringency was increased to select for the strongest binding phage (33 pM PSMA-His_6_). After rinsing to remove non-binding phages (TBS), bound phage were eluted under acidic conditions, neutralized, amplified and purified for the second round of screening. Individual phage clones were selected on Kanamycin and Ampicillin antibiotic plates and sequenced after the second and third rounds of screening.

### DNA sequencing

The phagemids of PSMA-bound phages were isolated (TRIzol reagent, Invitrogen, Carlsbad, CA), purified with (phenol/chloroform) and used as templates for DNA sequencing. PCR was used to amplify using the primer 5′-TGAATTTTCTGTATGAGG-3′ (reverse primer 3′-ACTTAAAAGACATACTCC-5′). Template amplification was done using BigDye Terminator (V3.1, Invitrogen/Applied Biosystems, Carlsbad, CA). The PCR reaction started at 98**°**C for 5 minutes followed by 30 cycles of amplification at 96**°**C for 15 seconds, 55**°**C for 15 seconds, 60**°**C for 4 minutes. The DNA sequences of phages were used to deduce the peptide sequence (Table S1). A GeneBank (BLAST) homology search was performed of consensus sequences that emerged from the screening to determine whether they are homologous to motifs present in human proteins.

### Cell Culture

The LNCaP and PC-3 human prostate cancer cell lines (American Type Culture Collection, Rockville, MD) were maintained in RPMI 1640 medium (Life Technologies, Grand Island, NY) containing 10% fetal bovine serum (FBS; Invitrogen/GIBCO, Carlsbad, CA), 100 U/ml penicillin, 100 µg/ml streptomycin in 5% CO_2_/95% air at 37**°**C.

### Determination of Cell Surface Binding and Internalization by Confocal Microscopy

The binding and cellular internalization study of phage clones and 5-FAM labeled peptides was performed as described previously [28]. Briefly, LNCaP and PC-3 cells (1×10^4^ cells/well) were grown on LabTek 8-chamber slides (Nunc Inc. Rochester, NY) overnight prior to the experiment. To determine the binding and internalization of phage clones, each clone (10^9^ cfu) was incubated with LNCaP or PC-3 cells adherent in chamber slides (37**°**C, 2 h). After rinsing (PBS), the cells were fixed and permeabilized (4% paraformaldehyde, 0.01% Triton-X, 15 min) and blocked (5% BSA, TBS). To visualize the surface bound and internalized phage clones, the fixed and permeabilized cells were treated with either biotinylated rabbit anti-Fd bacteriophage antibody and Alexa 488 conjugated to streptavidin, or rabbit monoclonal anti-PSMA antibody (primary antibody) with TRITC labeled donkey anti-Rabbit IgG (secondary antibody). To inhibit the specific binding of the phage clones, the cells were pre-incubated with PSMA-His_6_ (100 nM, 2 hours, 37**°**C) before adding the phage clones. To quantify the fluorescence, the relative fluorescence unit (rfu) in the LNCap cells was measured with the software in Olympus FV1000 confocal scanning microscope.. Regions of interest were drawn in ten cells to determine the rfu values.

To determine whether the discovered peptides from the third round of screening bound PSMA-positive LnCAP cells and co-internalized with PSMA, the 5-FAM labeled peptides (DMSO∶TBS 80∶20, v/v) were diluted in culture medium (1 µM) and incubated with adherent LNCaP cells in 8-well chamber slides (1 h, 37**°**C). After rinsing with PBS, the cells were fixed (4% paraformaldehyde, 15 min) and permeabilized (0.01% Triton-X, 15 min). Non-specific binding sites were blocked (5% BSA, TBS, 1 h), and then the cells were incubated with 1∶1000 rabbit monoclonal anti-PSMA antibody (primary antibody, 3% BSA, TBS, 1 h). After rinsing (PBS), the cells were incubated with 1∶1000 TRITC labeled donkey anti-Rabbit IgG (secondary antibody, 3% BSA, TBS, 1 h). After rinsing with PBS, the cells were mounted (50% glycerol, PBS), covered with coverslips, and sealed with nail polish. Confocal laser scanning microscopy (Olympus FV1000) was used to analyze all samples. We also used *z*-stack images to determine the extracellular and intracellular locations of the 5-FAM labeled peptides.

### AlphaScreen assay

The AlphaScreen competition binding assay was performed based on published procedures [29], [30], [31]. Briefly, to a 384-well, low flange, white flat bottom polystyrene NBS microplate (Corning, Tewksbury, MA), biotinylated peptide 563 (563-biotin, 5 µL, 600 nM), PSMA (10 µL, 30 nM) and buffer or peptide (various concentrations, 5 µL) were added. The mixture was incubated (ambient temperature, 1 h), and then nickel chelate acceptor beads (5 µL, 120 µg/mL) were added followed by a second incubation (dark, ambient temperature, 1 h). Streptavidin donor beads (5 µL, 120 µg/mL) were added for a final incubation (dark, ambient temperature, 30 m). The amount of bound 563-biotin to PSMA-His_6_ was quantified on a Synergy H4 hybrid multi-mode microplate reader (Biotek) using factory recommended settings. The final concentration for the donor/acceptor beads was 20 µg/mL, and the buffer used in this assay was 25 mM HEPES, 100 mM NaCl, pH 7.4. Data was analyzed by non-linear regression with GraphPad Prism.

### PSMA enzyme assay

The glutamic acid/glutamate released from the natural substrate of PSMA, NAAG (N-acetylaspartylglutamate), was analyzed by the fluorescence-based Amplex Red Glutamic Acid/Glutamate Oxidase assay kit (Invitrogen) as previously described [21], [22], [32], [33]. Briefly, in a 96-well half area black flat bottom polystyrene NBS microplate (Corning, Tewksbury, MA), PSMA-His_6_ (3 nM, 10 µL) and different concentrations of peptide 562 (monomer or tetramer, 20 µL) or buffer (TBS, 20 µL) were incubated (37**°**C, 30 min). Then NAAG (4 µM, 10 µL) was added to the peptide/PSMA-His_6_ mixture (37**°**C, 30 min). The enzymatic reaction was quenched (500 mM NaH_2_PO_4_/Na_2_HPO_4_, pH 7.4, 10 µL). The fluorescence signals generated from the Amplex working solution (50 µL) with the NAAG-liberated glutamate (37**°**C, 1 hour, light exclusion) were then quantified by the Synergy H4 hybrid multi-mode microplate reader (Ex 530 nm, Em 590 nm). Data was analyzed by non-linear regression with GraphPad Prism.

## Results and Discussion

### Recoveries of phage during panning

**Figure 1:**
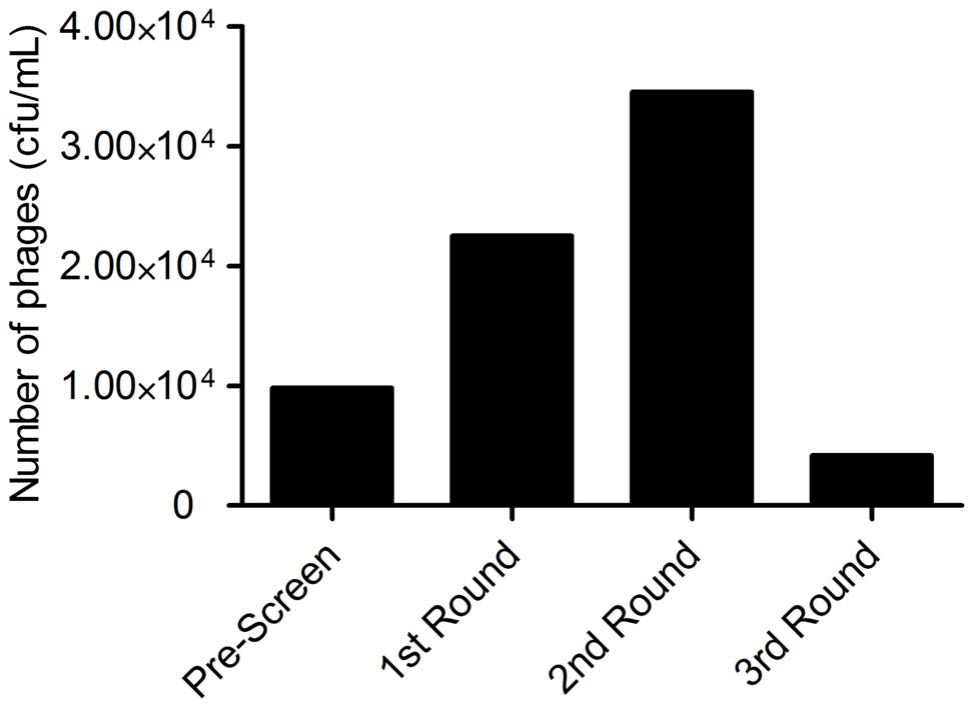
Recoveries from screening phage displayed libraries against the soluble ectodomain of PSMA.

A 15 amino acid fUSE5 phage display library was used as input phage to screen against poly-histidine tagged PSMA (PSMA-His_6_) immobilized in 6-well plates. This library had been previously depleted of phage that bound the endothelial cells of mouse and then re-amplified. After a depletion of the library of phage that bound non-specifically to the selection components consisting of BSA and anti-His_6_-Mab, the phage display library of 15-mers, was panned against PSMA-His_6_ that was captured by immobilized anti-His_6_-Mab. In rounds one and two the recovered phage was rising which is indicative of positive selection; however, the recovery decreased significantly when the concentration of PSMA-His_6_ was decreased from 42 nM to 33 pM from the second to third round of selection, respectively (Figure 1). The binding capacity of the plate was ∼10 nM in the capturing antibody, therefore we exceeded the binding capacity of the plate by ∼4-fold, assuming a high affinity of the anti-His_6_-Mab for the His_6_ tag. The very low concentration of PSMA (33 pM) was used to increase the stringency of specific binding to PSMA so that only the highest affinity peptides would be selected [34]. After the third round of selection, three strong consensus sequences emerged: GDHSPFT, SHFSVGS and EVPRLSLLAVFL, where the former sequence appeared three times and the latter two sequences appeared four times (Table 1). Consensus sequences suggest the presence of a defined binding motif whose affinity could be enhanced by key amino acid substitutions within or adjacent to the consensus sequence itself. A BLAST (NCBI) search algorithm for the sequences GDHSPFT, SHFSVGS and EVPRLSLLAVFL revealed a number of human proteins with motifs that have significant homology to the consensus sequences. However, there was no obvious correlation between them and possible binding partners for PSMA.

### Microscopy of phage clones

**Figure 2:**
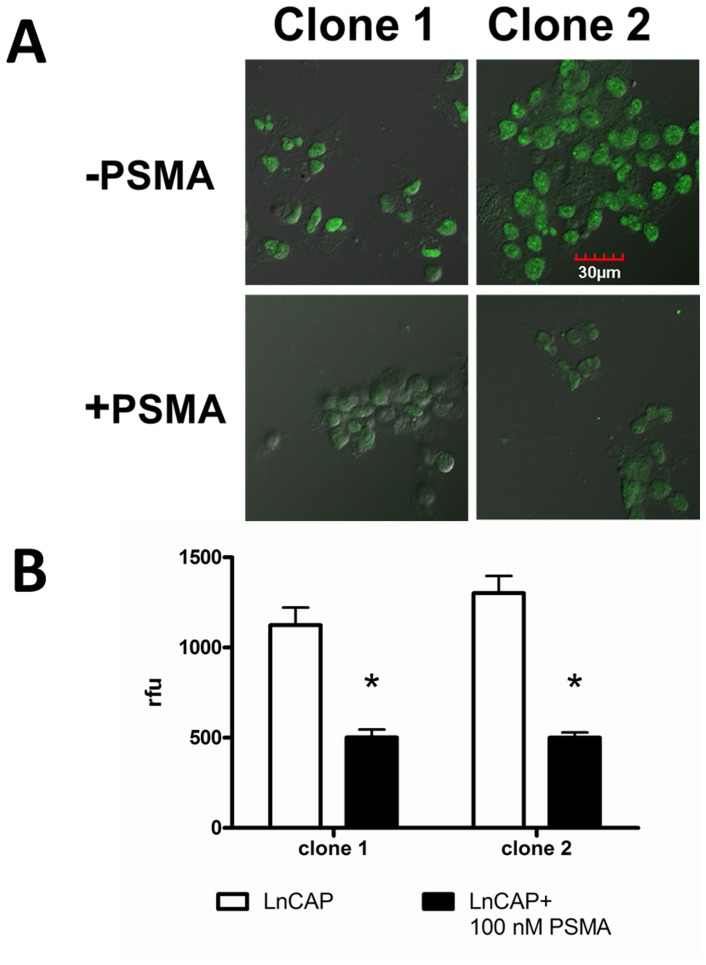
Immunofluorescence microscopy of phage clones from round 2 of panning. *(A)* PSMA (100 nM) inhibited the binding of Clone 1 (ARLSHRPSYLLVCA) and Clone 2 (GTAVASRVYSLHSLM) to PSMA-postive LNCaP cells, indicating that the uptake was receptor specific (scale is 30 µm). *(B)* Immunofluorescence microscopy of phage clones from round 2 of panning indicate that the panning process is biased toward PSMA binding (“*” denotes *P<0.05*).

**Figure 3:**
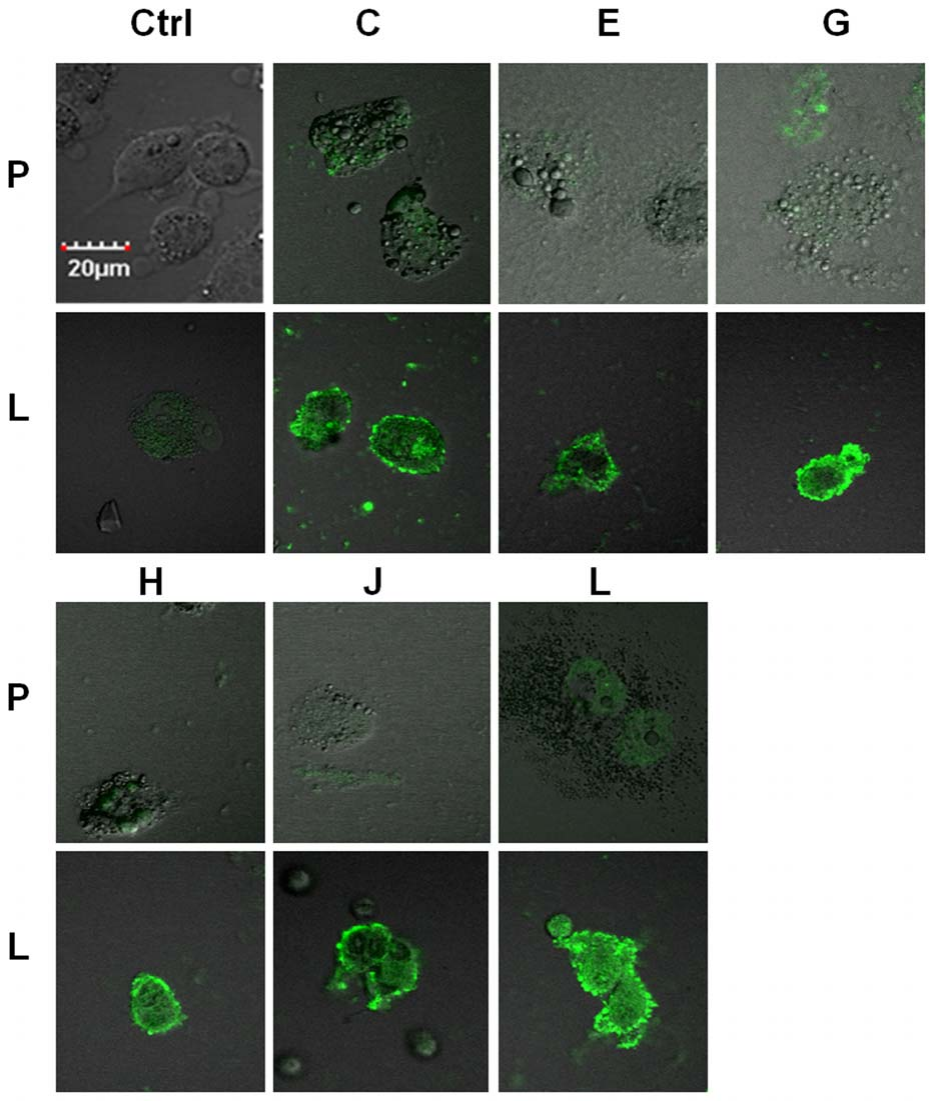
Immunofluorescence microscopy of phage clones from round 3 of panning. Randomly selected PSMA-targeted phage clones (C, E, G, H, J, L) from the third round of screening bound specifically to LNCaP cells (“L”), but not PC3 cells (“P”). While staining was preferentially bound at cell surface, there was evidence of internalization (scale 20 µm).

The phage displayed library was panned against soluble PSMA, which according to structural studies, exists predominately in the dimeric state and is fully functional in that state [35]. However, it was not known whether the soluble homodimer would be in a conformational state that would produce binding peptides that would also recognize PSMA naturally expressed in human cancer cells. To determine if the selected phage clones did recognize PSMA in its natural state, binding of the clones was visualized by confocal immuno-fluorescence microscopy on the human prostate cancer cells LNCaP (PSMA positive) and PC3 (PSMA negative). To verify that the selection was positively biased toward PSMA selection after round 2, eight clones were selected. Clone 1 (ARLSHRPSYLLVCA) appeared once and clone 2(GTAVASRVYSLHSLM) appeared seven times. The binding of clones 1 and 2 to PSMA was tested with LNCaP cells with and without an inhibitory concentration of PSMA-His_6_ (100 nM). The addition of soluble PSMA significantly reduced binding to the LNCaP cells for both clones (*P<0.05*, students *t*-test) indicating that the selection strategy had biased the library toward PSMA binding (Figure 2). After round 3 of selection, clones C, E, G, H, J, and L were tested for PSMA-mediated binding to LNCaP cells. These phage clones bound PSMA-positive LNCaP cells much more strongly than PSMA-negative PC3 cells utilizing the same input quantities for both cells (Figure 3). The staining is strong at the cell periphery where PSMA is localized. In some cases, vesicles are starting to appear on the cell periphery which is indicative of internalization, which has been previously observed with other PSMA targeted ligands [15]. This data indicate that the library is definitely biased toward PSMA binding and the tested clones do indeed bind PSMA in its natural state on human cancer cells. At this point, the panning process was stopped and the displayed peptide sequences were tested for further development as PSMA ligands.

### Co-localization of the targeted peptides and PSMA on the surface of LNCaP cells

**Figure 4:**
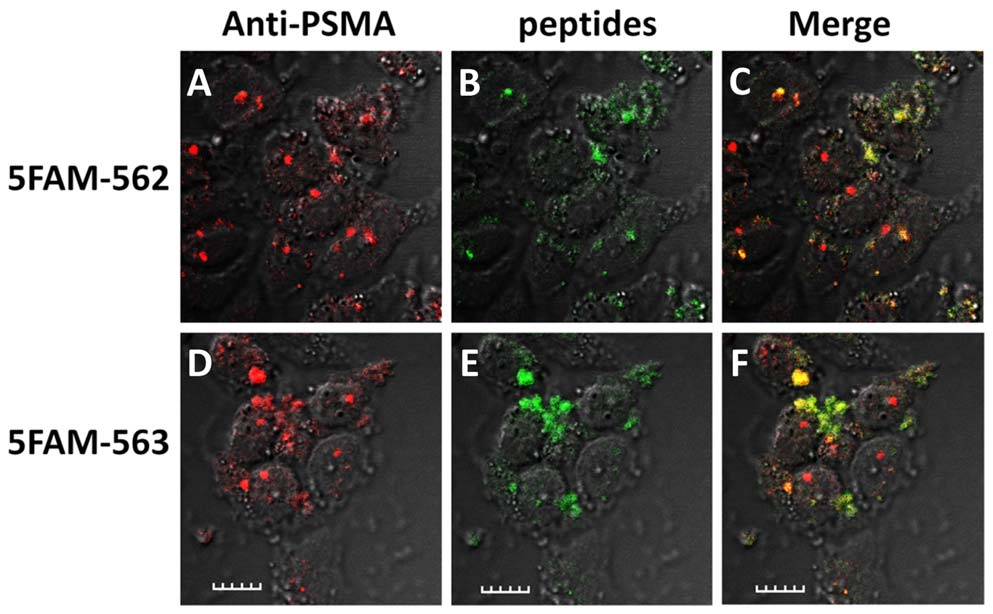
Binding and internalization of 5FAM-562 and 5FAM-563 in LNCaP cells. 5FAM-562 (1 µM, *A–C*, green) and 5FAM-563 (1 µM , *D–F*, green) bound and internalized in LNCaP cells and co-localized with PSMA in intracellular vesicles visualized with anti-PSMA-Mab (red, scale is 20 µm).

To further address the specificity and affinity of the discovered peptides toward PSMA-binding among the selected phages in the third round of screening, a total of 13 phage clones were selected and their phagemids DNA were isolated, sequenced and deduced to protein sequences, which were used as templates for syntheses of the 15-mer peptides. Three consensus sequences, -SHSFSVGSG-, -GDHSPFT- and -EVPRLSLLAVFL- were found in the screening (Table 1). Two peptides containing a consensus sequence, SHSFSVGSGDHSPFT (562) and GRFLTGGTGRLLRIS (563), were synthesized and conjugated with 5-carboxyl fluorescein (5FAM) on the N-terminal amine to determine whether they would induce internalization in LNCaP cells (Figure 4). These results show strong co-staining between the 5FAM-562 and 5FAM-563 and PSMA located in intra- cellular vesicular compartments within the LNCaP cells indicating that both peptides bind PSMA and induce internalization which would be an important attribute for molecular imaging (Figure 4) [12], [13], [14]. Previously, monoclonal antibodies and small molecule PSMA-inhibitors have been shown to be internalized into cells, mediated by PSMA [11], [36]. On phage, the peptides are connected C-terminally to the phage and it is possible that modifications at the N-terminus could reduce binding affinity; however, if there was any reduction in affinity, it was not great enough to completely inhibit binding. These results indicate that the peptides 562 and 563 discovered through phage display library are indeed highly-specific PSMA-binding peptides.

### Binding Affinities by AlphaScreen and PSMA enzyme assays

AlphaScreen is a bead-based non-radioactive homogenous proximity assay and has been used to study both strong (∼nM K_D_) and weak (∼µM K_D_) interactions between biomolecules. Because AlphaScreen is a homogenous assay, it is of particular value in evaluating peptides with putative weak affinities that are likely to be discovered from naïve libraries. In previous work, the K_D_-values derived from AlphaScreen assay were consistent with those reported using other methods such as radio-ligand binding assay, fluorescence polarization assay and surface plasmon resonance (SPR) assays [30], [31], [37], [38]. In our study, the biotinylated peptide 563 bound the streptavidin donor bead and the PSMA-His_6_ bound the nickel chelate acceptor bead. Only when the peptide binds to PSMA (thus bringing the two beads close to each other), can the AlphaScreen (emission) signal be generated, in which case, the singlet oxygen generated by the excitation at 680 nm on the donor bead can transfer energy to the adjacent acceptor bead and subsequently produce an emission signal at 520–620 nm.

**Figure 5:**
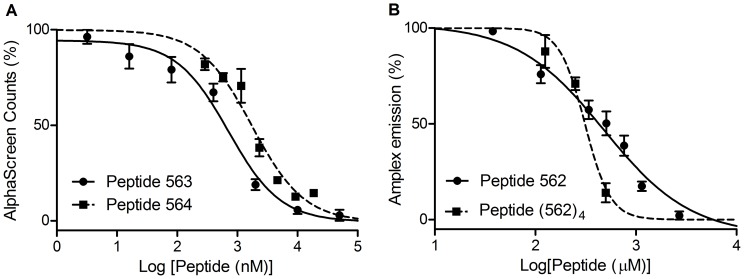
Evaluation of binding affinities between phage display-discovered peptides and PSMA. (*A*) AlphaScreen assay in which Peptide 563 (filled circles) or Peptide 564 (filled squares) competed with biotinylated peptide 563 for binding to PSMA. Data was fitted to generate IC_50_ curves for Peptide 563 (solid line) and Peptide 564 (dashed line). (*B*) Fluorescence-based PSMA enzyme inhibition assay in which Peptide 562 monomer (filled circles) or Peptide 562 tetramer (filled squares) inhibited the hydrolysis of NAAG (substrate) by PSMA. Data was fit to generate IC_50_ curves for Peptide 562 monomer (solid line) and Peptide 562 tetramer (dashed line).

To test the affinity of peptides discovered by phage display, peptides 562, 563, 564, and 563-biotin were synthesized and purified by RP-HPLC and tested for affinity in either AlphaScreen or in a PSMA inhibition assay. The peptides bearing the relatively hydrophobic consensus sequence EVPRLSLLAVFL were synthesized, but due to the extremely limited aqueous solubility they were not tested. In the AlphaScreen binding assay, peptide 563, 564, and 565 were used to displace biotinylated peptide 563 with concomitant loss in signal. The IC_50_ values were used to approximate the K_D_ between the displacing peptides and PSMA (Figure 5A). Peptide 563 (IC_50_ = 708 nM, 95%) and 564 (IC_50_ = 1.72 µM, 95% confidence intervals: 1.09–2.71 µM) displace 563-biotin in a log dose-dependent manner. In control experiments peptide 562 generated high background counts due to nickel binding (through histidines) properties and was thus not suited for analysis by AlphaScreen but did inhibit the PSMA enzymatic reaction (against 1 µM NAAG) and gave an IC_50_ value of 661 µM (95% confidence intervals: 426–1024 µM, Figure 5B). Utilizing the Cheng-Prusoff equation, K**_I_** = IC**_50_**/(1 + [S]/K**_M_**) and K**_M_**  = 0.13 µM, a K**_I_** value of 76 µM can be computed [39]. Peptide 565 was relatively insoluble in aqueous buffers and was not active at a concentration of 4.83 µM in AlphaScreen or 14.5 µM in the PSMA enzyme inhibition assay. With limited water solubility, peptide 564, at a concentration of 55.5 µM, could not inhibit the PSMA enzymatic reaction. Interestingly, even with a concentration of 1.07 mM, peptide 563 could not inhibit the PSMA enzymatic reaction under our test conditions.

**Figure 6:**
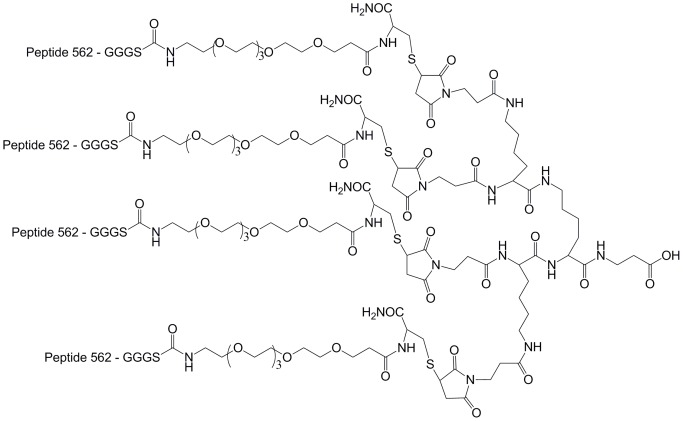
Structure of the Peptide 562 tetramer.

Quantitative studies of the affinity between peptide ligands discovered by phage display and PSMA have only been investigated by the PSMA enzyme inhibition assay [32], [40], [41], [42]. To the best of our knowledge, this is the first investigation of affinities of peptide ligands for PSMA that are not based on the inhibition of the enzymatic activity of PSMA. The AlphaScreen technology is advantageous in assessing the affinity between the peptide ligand and PSMA as it does not depend on the inhibition of the PSMA enzymatic activity and it only requires very low concentration (∼nM) of PSMA. In fact, peptide 563 showed that it can bind PSMA with relatively high affinity (sub- μM K_D_) but cannot inhibit the PSMA enzymatic reaction. For one of the peptides which does inhibit the PSMA enzymatic activity, peptide 562, the IC_50_ value (IC_50_ = 661 µM) is relatively higher than the IC_50_ of aforementioned peptide ligand discovered by Aggarwal et al. (IC_50_ = 23 µM). The Peptide-562-tetramer (562_4_, Figure 6) only gave 2-fold improvement with an approximate IC_50_ value of 311 µM (95% confidence intervals: 267–363 µM, Figure 5B). In comparison, both Aggarwal *et al*. and Banerjee *et al*. [43] showed ∼10-fold improvement on the PSMA enzyme inhibitory activity with their multimeric PSMA-binding ligands when comparing to the monomeric ones. Aggarwal *et al*. [32] attributed such enhancement to the β-hairpin-like secondary structure of the dimeric peptide ligand they made while Banerjee *et al*. attributed it to the optimized long-linker (20 Å) incorporated in their bivalent small-molecule-based ligand. For the sequences discovered in this work, further studies on the secondary structure of those sequences might elucidate the binding mode of residues within the sequence.

Notably, the PSMA enzyme-inhibitory peptide 562 and PSMA enzyme non-inhibitory peptide 563 may bind to PSMA at different surface areas. Peptide 562, SHSFSVGSGDHSPFT, is hydrophilic relative to the very hydrophobic peptide sequence, EVPRLSLLAVFL, and these two peptides may bind to PSMA at different surface areas as well. Thus a potential strategy to increase affinity is a multimeric peptide ligand that would incorporate both hydrophobic and hydrophilic sequences such that two or more sequences could work synergistically for binding PSMA with high affinity and high specificity. This strategy to target different surface areas of the protein of interest has been applied in the fields of phage displayed peptide libraries [44], [45], OBOC (one-bead-one-compound) peptide libraries [46] and peptoid microarrays [47].

## Conclusions

Through panning a phage displayed peptide library against soluble PSMA, we anticipated the emergence of peptide sequences for further development into high affinity ligands for PSMA. While the increase in stringency in the last step of panning did not produce peptides of high affinity, three strong consensus sequences did emerge, GDHSPFT, SHFSVGS and EVPRLSLLAVFL in addition to several sequences that were not in consensus. One interesting trend in the sequences was peptides that bound PSMA but did not inhibit the enzymatic activity of the PSMA. The next steps for these sequences would be affinity maturation. A ten-fold improvement of sequence GRFLTGGTGRLLRIS, with its IC_50_ value of ∼700 nM would result in a peptide with affinity that would be suitable for the non-invasive diagnosis and staging of PCa when labeled with the appropriate isotope for PET. This sequence may even be suitable for conjugation to nanoparticles for therapy of prostate cancer through the enhancement of affinity through avidity.

## Supporting Information

Table S1 DNA sequences of PSMA-targeted clones.
(DOC)

## References

[1] Nam RK, Oliver TK, Vickers AJ, Thompson I, Kantoff PW, et al (2012) Prostate-specific antigen test for prostate cancer screening: American Society of Clinical Oncology provisional clinical opinion. Journal of oncology practice/American Society of Clinical Oncology 8: 315–317. 10.1200/JOP.2012.000715 PMC3439233 23277770

[2] Basch E, Oliver TK, Vickers A, Thompson I, Kantoff P, et al (2012) Screening for prostate cancer with prostate-specific antigen testing: American Society of Clinical Oncology Provisional Clinical Opinion. Journal of clinical oncology : official journal of the American Society of Clinical Oncology 30: 3020–3025. 22802323 10.1200/JCO.2012.43.3441 PMC3776923

[3] Hessels D, Verhaegh GW, Schalken JA, Witjes JA (2004) Applicability of biomarkers in the early diagnosis of prostate cancer. Expert Rev Mol Diagn 4: 513–526. 15225099 10.1586/14737159.4.4.513

[4] Roobol MJ, Kranse R, de Koning HJ, Schroder FH (2004) Prostate-specific antigen velocity at low prostate-specific antigen levels as screening tool for prostate cancer: results of second screening round of ERSPC (ROTTERDAM). Urology 63: 309–313; discussion 313–305. 14972478 10.1016/j.urology.2003.09.083

[5] Thompson IM, Pauler DK, Goodman PJ, Tangen CM, Lucia MS, et al (2004) Prevalence of prostate cancer among men with a prostate-specific antigen level < or = 4.0 ng per milliliter. N Engl J Med 350: 2239–2246. 15163773 10.1056/NEJMoa031918

[6] Minner S, Wittmer C, Graefen M, Salomon G, Steuber T, et al (2011) High level PSMA expression is associated with early PSA recurrence in surgically treated prostate cancer. Prostate 71: 281–288. 20809553 10.1002/pros.21241

[7] Xiao Z, Adam BL, Cazares LH, Clements MA, Davis JW, et al (2001) Quantitation of serum prostate-specific membrane antigen by a novel protein biochip immunoassay discriminates benign from malignant prostate disease. Cancer Res 61: 6029–6033. 11507047

[8] Ghosh A, Heston WD (2004) Tumor target prostate specific membrane antigen (PSMA) and its regulation in prostate cancer. Journal of cellular biochemistry 91: 528–539. 14755683 10.1002/jcb.10661

[9] Morris MJ, Pandit-Taskar N, Divgi CR, Bender S, O'Donoghue JA, et al (2007) Phase I evaluation of J591 as a vascular targeting agent in progressive solid tumors. Clin Cancer Res 13: 2707–2713. 17473203 10.1158/1078-0432.CCR-06-2935

[10] Chang SS, O'Keefe DS, Bacich DJ, Reuter VE, Heston WD, et al (1999) Prostate-specific membrane antigen is produced in tumor-associated neovasculature. Clin Cancer Res 5: 2674–2681. 10537328

[11] Liu H, Moy P, Kim S, Xia Y, Rajasekaran A, et al (1997) Monoclonal antibodies to the extracellular domain of prostate-specific membrane antigen also react with tumor vascular endothelium. Cancer Res 57: 3629–3634. 9288760

[12] Morris MJ, Divgi CR, Pandit-Taskar N, Batraki M, Warren N, et al (2005) Pilot trial of unlabeled and indium-111-labeled anti-prostate-specific membrane antigen antibody J591 for castrate metastatic prostate cancer. Clin Cancer Res 11: 7454–7461. 16243819 10.1158/1078-0432.CCR-05-0826

[13] Milowsky MI, Nanus DM, Kostakoglu L, Vallabhajosula S, Goldsmith SJ, et al (2004) Phase I trial of yttrium-90-labeled anti-prostate-specific membrane antigen monoclonal antibody J591 for androgen-independent prostate cancer. J Clin Oncol 22: 2522–2531. 15173215 10.1200/JCO.2004.09.154

[14] Bander NH, Milowsky MI, Nanus DM, Kostakoglu L, Vallabhajosula S, et al (2005) Phase I trial of 177lutetium-labeled J591, a monoclonal antibody to prostate-specific membrane antigen, in patients with androgen-independent prostate cancer. J Clin Oncol 23: 4591–4601. 15837970 10.1200/JCO.2005.05.160

[15] Liu H, Rajasekaran AK, Moy P, Xia Y, Kim S, et al (1998) Constitutive and antibody-induced internalization of prostate-specific membrane antigen. Cancer Res 58: 4055–4060. 9751609

[16] Sodee DB, Faulhaber PF, Nelson AD, Bakale G (2004) The prognostic significance of indium-111-capromab penetide. J Clin Oncol 22: 379–380; author reply 380–371. 14722051 10.1200/JCO.2004.99.163

[17] Banerjee SR, Foss CA, Castanares M, Mease RC, Byun Y, et al (2008) Synthesis and evaluation of technetium-99m- and rhenium-labeled inhibitors of the prostate-specific membrane antigen (PSMA). Journal of medicinal chemistry 51: 4504–4517. 18637669 10.1021/jm800111u PMC3336105

[18] Banerjee SR, Pullambhatla M, Byun Y, Nimmagadda S, Green G, et al (2010) 68Ga-labeled inhibitors of prostate-specific membrane antigen (PSMA) for imaging prostate cancer. Journal of medicinal chemistry 53: 5333–5341. 20568777 10.1021/jm100623e PMC3341619

[19] Chen Y, Foss CA, Byun Y, Nimmagadda S, Pullambhatla M, et al (2008) Radiohalogenated prostate-specific membrane antigen (PSMA)-based ureas as imaging agents for prostate cancer. J Med Chem 51: 7933–7943. 19053825 10.1021/jm801055h PMC2631656

[20] Chen Y, Pullambhatla M, Foss CA, Byun Y, Nimmagadda S, et al (2011) 2-(3-{1-Carboxy-5-[(6-[18F]fluoro-pyridine-3-carbonyl)-amino]-pentyl}-ureido)-pen tanedioic acid, [18F]DCFPyL, a PSMA-based PET imaging agent for prostate cancer. Clinical cancer research : an official journal of the American Association for Cancer Research 17: 7645–7653. 22042970 10.1158/1078-0432.CCR-11-1357 PMC3243762

[21] Chen Y, Dhara S, Banerjee SR, Byun Y, Pullambhatla M, et al (2009) A low molecular weight PSMA-based fluorescent imaging agent for cancer. Biochemical and Biophysical Research Communications 390: 624–629. 19818734 10.1016/j.bbrc.2009.10.017 PMC2787846

[22] Humblet V, Lapidus R, Williams LR, Tsukamoto T, Rojas C, et al (2005) High-affinity near-infrared fluorescent small-molecule contrast agents for in vivo imaging of prostate-specific membrane antigen. Molecular imaging 4: 448–462. 16285907 10.2310/7290.2005.05163

[23] Haubner R, Decristoforo C (2011) Radiotracer II: Peptide-Based Radiopharmaceuticals. In: Kiessling F, Pichler BJ, editors. Small Animal Imaging: Springer Berlin Heidelberg. pp. 247–266.

[24] Noren KA, Noren CJ (2001) Construction of high-complexity combinatorial phage display peptide libraries. Methods 23: 169–178. 11181036 10.1006/meth.2000.1118

[25] Newton JR, Kelly KA, Mahmood U, Weissleder R, Deutscher SL (2006) In vivo selection of phage for the optical imaging of PC-3 human prostate carcinoma in mice. Neoplasia 8: 772–780. 16984734 10.1593/neo.06331 PMC1584300

[26] Li SZ, McGuire MJ, Lin M, Liu YH, Oyama T, et al (2009) Synthesis and characterization of a high-affinity alpha(v)beta(6)-specific ligand for in vitro and in vivo applications. Molecular Cancer Therapeutics 8: 1239–1249. 19435868 10.1158/1535-7163.MCT-08-1098 PMC4053473

[27] Barbas III CF, Buron DR, Silverman GJ, editors(2001) Phage Display: A Laboratory Manual. 1st ed. Cold Spring Harbor: Cold Spring Harbor Laboratory Press. 736 p.

[28] Shen D, Liang K, Ye Y, Tetteh E, Achilefu S (2007) Modulation of nuclear internalization of Tat peptides by fluorescent dyes and receptor-avid peptides. FEBS Lett 581: 1793–1799. 17416362 10.1016/j.febslet.2007.03.067 PMC1934384

[29] Yi F, Zhu P, Southall N, Inglese J, Austin CP, et al (2009) An AlphaScreen (TM)-Based High-Throughput Screen to Identify Inhibitors of Hsp90-Cochaperone Interaction. Journal of Biomolecular Screening 14: 273–281. 19211782 10.1177/1087057108330114 PMC3066041

[30] Quinn AM, Bedford MT, Espejo A, Spannhoff A, Austin CP, et al (2010) A homogeneous method for investigation of methylation-dependent protein-protein interactions in epigenetics. Nucleic Acids Research 38. 10.1093/nar/gkp899 PMC2811012 19897549

[31] Wigle TJ, Herold JM, Senisterra GA, Vedadi M, Kireev DB, et al (2010) Screening for Inhibitors of Low-Affinity Epigenetic Peptide-Protein Interactions: An AlphaScreen (TM)-Based Assay for Antagonists of Methyl-Lysine Binding Proteins. Journal of Biomolecular Screening 15: 62–71. 20008125 10.1177/1087057109352902

[32] Aggarwal S, Singh P, Topaloglu O, Isaacs JT, Denmeade SR (2006) A dimeric peptide that binds selectively to prostate-specific membrane antigen and inhibits its enzymatic activity. Cancer Research 66: 9171–9177. 16982760 10.1158/0008-5472.CAN-06-1520

[33] Banerjee SR, Pullambhatla M, Byun Y, Nimmagadda S, Foss CA, et al (2011) Sequential SPECT and Optical Imaging of Experimental Models of Prostate Cancer with a Dual Modality Inhibitor of the Prostate-Specific Membrane Antigen. Angewandte Chemie-International Edition 50: 9167–9170. 21861274 10.1002/anie.201102872 PMC3192196

[34] Yu J, Smith GP (1996) Affinity maturation of phage-displayed peptide ligands. Methods in enzymology 267: 3–27. 8743307 10.1016/s0076-6879(96)67003-7

[35] Davis MI, Bennett MJ, Thomas LM, Bjorkman PJ (2005) Crystal structure of prostate-specific membrane antigen, a tumor marker and peptidase. Proceedings of the National Academy of Sciences of the United States of America 102: 5981–5986. 15837926 10.1073/pnas.0502101102 PMC556220

[36] Hillier SM, Maresca KP, Femia FJ, Marquis JC, Foss CA, et al (2009) Preclinical evaluation of novel glutamate-urea-lysine analogues that target prostate-specific membrane antigen as molecular imaging pharmaceuticals for prostate cancer. Cancer Research 69: 6932–6940. 19706750 10.1158/0008-5472.CAN-09-1682 PMC4114247

[37] Lazar GA, Dang W, Karki S, Vafa O, Peng JS, et al (2006) Engineered antibody Fc variants with enhanced effector function. Proceedings of the National Academy of Sciences of the United States of America 103: 4005–4010. 16537476 10.1073/pnas.0508123103 PMC1389705

[38] Pawlyk AC, Cassel JA, Blass BE, Reitz AB (2010) Development of a novel non-radiometric assay for nucleic acid binding to TDP-43 suitable for high-throughput screening using AlphaScreen technology. Society for Neuroscience Abstract Viewer and Itinerary Planner 40. 10.1177/1087057110382778 PMC3426361 20855563

[39] Mesters JR, Barinka C, Li W, Tsukamoto T, Majer P, et al (2006) Structure of glutamate carboxypeptidase II, a drug target in neuronal damage and prostate cancer. The EMBO journal 25: 1375–1384. 16467855 10.1038/sj.emboj.7600969 PMC1422165

[40] Arter JA, Diaz JE, Donavan KC, Yuan T, Penner RM, et al (2012) Virus-Polymer Hybrid Nanowires Tailored to Detect Prostate-Specific Membrane Antigen. Analytical Chemistry 84: 2776–2783. 22339784 10.1021/ac203143y PMC3956303

[41] Lupold SE, Rodriguez R (2004) Disulfide-constrained peptides that bind to the extracellular portion of the prostate-specific membrane antigen. Mol Cancer Ther 3: 597–603. 15141017

[42] Zhu ZY, Zhong CP, Xu WF, Lin GM, Ye GQW, et al (1999) PSMA mimotope isolated from phage displayed peptide library can induce PSMA specific immune response. Cell Research 9: 271–280. 10628836 10.1038/sj.cr.7290026

[43] Banerjee SR, Pullambhatla M, Shallal H, Lisok A, Mease RC, et al (2011) A Modular Strategy to Prepare Multivalent Inhibitors of Prostate-Specific Membrane Antigen (PSMA). Oncotarget 2: 1244–1253. 22207391 10.18632/oncotarget.415 PMC3282081

[44] Diehnelt CW, Shah M, Gupta N, Belcher PE, Greving MP, et al (2010) Discovery of High-Affinity Protein Binding Ligands - Backwards. Plos One 5. 10.1371/journal.pone.0010728 PMC2873402 20502719

[45] Kim S, Kim D, Jung HH, Lee I-H, Kim JIL, et al (2012) Bio-Inspired Design and Potential Biomedical Applications of a Novel Class of High-Affinity Peptides. Angewandte Chemie-International Edition 51: 1890–1894. 22271427 10.1002/anie.201107894

[46] Agnew HD, Rohde RD, Millward SW, Nag A, Yeo W-S, et al (2009) Iterative In Situ Click Chemistry Creates Antibody-like Protein-Capture Agents. Angewandte Chemie-International Edition 48: 4944–4948. 19301344 10.1002/anie.200900488 PMC3716464

[47] Lim H-S, Reddy MM, Xiao X, Wilson J, Wilson R, et al (2009) Rapid identification of improved protein ligands using peptoid microarrays. Bioorganic & Medicinal Chemistry Letters 19: 3866–3869. 19380225 10.1016/j.bmcl.2009.03.153 PMC4452005

