# Supplementary material for: Evaluation of Phage Display Discovered Peptides as Ligands for Prostate-Specific Membrane Antigen (PSMA)
Source: PLoS One. 2013 Jul 25;8(7):e68339. doi: 10.1371/journal.pone.0068339 (PMC3723849; doi:10.1371/journal.pone.0068339)
Supplement: Table S1 — DNA sequences of PSMA-targeted clones. (DOC) [file pone.0068339.s001.doc]

Table S1: DNA sequences of PSMA-targeted clones

clone B:

5'-CTT CCC ATC TTT AAG GTT GAT TTC GGT GAT CAT TCT CCT TTC ACT

**Leu Pro Ile Phe lys Val Asp Phe Gly Asp His Ser Pro Phe Thr**

clone C:

**5’-** GCT AGA ATG TTC CTC CTA TTT TTA ATG AGG TGT ATC GGA TGC TAT**-3’**

**Ala Arg Met Phe Leu Leu Phe Leu Met Arg cys Ile Gly cys Tyr**

clone D and H:

5'-TCT CAT TCT TTT TCT GTT GGT TCT GGT GAT CAT TCT CCT TTC ACT-3’

**Ser His Ser Phe Ser Val Gly Ser Gly Asp His Ser Pro Phe Thr**

clone E and Clone F:

5’TCT CAT TCT TTT TCT GTT GGT TCT GGT TCT AAT CAT TCT CCT TTG

**Ser His Ser Phe Ser Val Gly Ser Gly Ser Asn His Ser Pro_Leu**

clone G

5’ TTG TCA TTC TTT TCC TGT TGG TTA AGG AGA TCA TTC TCT TTG ACT 3’

**Leu Ser Phe Phe Ser Cys Trp Leu Arg Arg Ser Phe Ser Leu Thr**

clone I:

5'-GAG GTG CCT CGT CTT TCG TTG CTT GCT GTG TCT CTT GTG CTA ATG

**Glu Val Pro Arg Leu Ser Leu Leu Ala Val Phe Leu Val Val Met**

clone J:

5'-GAG GTG CCT CGT CTT TCG TTG CTT GCT GTG TCT CTG TGC AAT GGG

**Glu Val Pro Arg Leu Ser Leu Leu Ala Val Phe Leu cys Asn Gly**

clone K:

5'-GAG GTG CCT CGT CTT TCG TTG CTT GCT GTG TCT CTT GTT GCT AAT

**Glu Val Pro Arg Leu Ser Leu Leu Ala Val Phe Leu Val Ala Asn**

clone L:

5'-GAG GTG CCT CGT CTT TCG TTG CTT GCT GTG TCT CTT GTT GCT AAT

**Glu Val Pro Arg Leu Ser Leu Leu Ala Val Phe leu Val Ala Asn**

clone M:

**5’**GGG AGA TTT TTA ACG GGG GGT ACA GGT CGC CTC CTG AGG ATC TCC

**Gly Arg Phe Leu Thr Gly Gly Thr Gly Arg Leu Leu Arg Ile Ser**
